# The Stereotypic Response of the Pulmonary Vasculature to Respiratory Viral Infections: Findings in Mouse Models of SARS-CoV-2, Influenza A and Gammaherpesvirus Infections

**DOI:** 10.3390/v15081637

**Published:** 2023-07-27

**Authors:** Simon De Neck, Rebekah Penrice-Randal, Jordan J. Clark, Parul Sharma, Eleanor G. Bentley, Adam Kirby, Daniele F. Mega, Ximeng Han, Andrew Owen, Julian A. Hiscox, James P. Stewart, Anja Kipar

**Affiliations:** 1 Laboratory for Animal Model Pathology, Vetsuisse Faculty, Institute of Veterinary Pathology, University of Zurich, 8057 Zurich, Switzerland; simon.deneck@uzh.ch; 2 Department of Infection Biology & Microbiomes, Institute of Infection, Veterinary and Ecological Sciences, University of Liverpool, Liverpool L3 3RF, UK; r.penrice-randal@liverpool.ac.uk (R.P.-R.); jordan.clark@mssm.edu (J.J.C.); parul.sharma@liverpool.ac.uk (P.S.); e.bentley@liverpool.ac.uk (E.G.B.); adam.kirby@liverpool.ac.uk (A.K.); d.f.mega@liverpool.ac.uk (D.F.M.); ximeng.han@ndm.ox.ac.uk (X.H.); julianh@liverpool.ac.uk (J.A.H.); j.p.stewart@liverpool.ac.uk (J.P.S.); 3 Centre of Excellence in Long-Acting Therapeutics (CELT), Department of Pharmacology and Therapeutics, University of Liverpool, Liverpool L3 3RF, UK; aowen@liverpool.ac.uk; 4 Department of Basic Veterinary Sciences, Faculty of Veterinary Medicine, University of Helsinki, 00790 Helsinki, Finland

**Keywords:** severe acute respiratory syndrome coronavirus 2, influenza A virus, murine gammaherpesvirus 68, mouse models, lung, vasculitis

## Abstract

The respiratory system is the main target of severe acute respiratory syndrome coronavirus 2 (SARS-CoV-2), the cause of coronavirus disease 19 (COVID-19) where acute respiratory distress syndrome is considered the leading cause of death. Changes in pulmonary blood vessels, among which an endothelialitis/endotheliitis has been particularly emphasized, have been suggested to play a central role in the development of acute lung injury. Similar vascular changes are also observed in animal models of COVID-19. The present study aimed to determine whether the latter are specific for SARS-CoV-2 infection, investigating the vascular response in the lungs of mice infected with SARS-CoV-2 and other respiratory viruses (influenza A and murine gammaherpesvirus) by in situ approaches (histology, immunohistology, morphometry) combined with RNA sequencing and bioinformatic analysis. Non-selective recruitment of monocytes and T and B cells from larger muscular veins and arteries was observed with all viruses, matched by a comparable transcriptional response. There was no evidence of endothelial cell infection in any of the models. Both the morphological investigation and the transcriptomics approach support the interpretation that the lung vasculature in mice mounts a stereotypic response to alveolar and respiratory epithelial damage. This may have implications for the treatment and management of respiratory disease in humans.

## 1. Introduction

Infection with the betacoronavirus severe acute respiratory syndrome coronavirus 2 (SARS-CoV-2) is the cause of coronavirus disease 19 (COVID-19). The clinical presentation of SARS-CoV-2 infection can vary substantially, ranging from asymptomatic to severe COVID-19 and death (reviewed in [1]); however, in the majority of affected individuals it does not go beyond flu-like symptoms [2] as they are also seen with influenza [3]. The main target of SARS-CoV-2 is the respiratory system and the lungs represent the primarily affected organs in patients with severe COVID-19 [2,4]. Pulmonary affliction, in the form of acute respiratory distress syndrome (ARDS), is considered as the leading cause of death. Histologically, it is characterized by diffuse alveolar damage (DAD), with edema, hemorrhage, hyaline membranes and pneumocyte degeneration [5]. A recent review based on post mortem examinations of fatal COVID-19 cases described progressive DAD, characterized by an acute and an organizing phase, as one of three major, non-exclusive patterns of lesions, with bronchopneumonia, as a consequence of secondary bacterial or fungal infection, and tissue thrombosis representing the other two [4]. Other authors introduced a four-stage DAD concept, with an early (days 0–1), an exudative (days 1–7), an organizing (one to several weeks) DAD and a fibrotic (weeks to months) stage that can coexist in the lungs [2].

The histological examination of autopsied cases provided evidence that fatal pulmonary disease in COVID-19 is also associated with vascular alterations; thrombosis in large and intermediate-sized vessels with endothelial damage and platelet–fibrin thrombi in small arteries and/or capillaries, sometimes associated with vascular and perivascular inflammatory infiltration, have been reported in variable combinations [6,7,8,9,10,11,12,13]; reviewed in [2]. A variety of terms have been applied when leukocytes were found within vessel walls, including vasculitis [8,9], capillaritis [13] and endothelialitis (endotheliitis) [6,7,10,12,13]. With time, endothelialitis (endotheliitis) has been promoted as a characteristic pathological feature of SARS-CoV-2 infection of the lung; it has also been applied in animal models of the disease, in particular the Syrian hamster [12,14,15,16,17,18,19] where a detailed histopathological examination outlined four types of vascular lesions, i.e., endothelialitis, endothelial hypertrophy, vasculitis and mural vascular wall degeneration [18]. Hence, it has been suggested that the vascular lesions play an important role in the pathogenesis of COVID-19 and are central in the development of acute lung injury [2]. Indeed, a review stated that some authors considered COVID-19 as an angiocentric disease [5]. Other reports have also described vascular changes (thrombosis, (peri)vascular infiltration, cell death) in other organs, such as the brain, heart, kidneys, liver and small intestine [6,20,21,22]. This raises the question as to whether SARS-CoV-2 targets vascular endothelial cells. Indeed, there are initial publications that report the presence of the virus in the endothelium [6,7,11,13]. Since then, controversial results have been published and the question has been under substantial debate, both for natural human infections and in animal models. While some authors have reported the presence of the virus/viral elements within endothelial cells, others did not find any evidence of endothelial cell infection [6,7,9,13,15,18,19,23,24,25,26,27,28]. In addition, a recent review came to the conclusion that human endothelial cells are resistant to SARS-CoV-2 infection, citing in vitro studies; this led to the hypothesis that endothelial injury in COVID-19 is the result of an indirect mechanism involving an exaggerated local inflammatory reaction and systemic immune response [29].

For other respiratory viral infections, vascular changes are rarely discussed [30,31], although vasculitis and endothelial injury have been reported in the lungs as well as other organs of patients infected with SARS-CoV [32,33,34]. However, in previous studies using murine models of influenza A virus (IAV) and gammaherpesvirus infections, we and others described vasculitis and perivascular leukocyte infiltrates as common features but did not go into substantial detail or mention endothelialitis-like vascular inflammatory infiltrations, which would not contradict the assumption that the latter is a phenomenon exclusive to SARS-CoV-2 infection. The present study aimed to address this concept and represents a detailed comparative investigation of the vascular changes observed in established murine models of respiratory viral infections with differences in pathogenesis and the extent of pulmonary damage, i.e., SARS-CoV-2, IAV and murine gammaherpesvirus 68 [35,36].

## 2. Materials and Methods

### 2.1. Cell Culture and Viruses

SARS-CoV-2 viruses: A Pango lineage B strain of SARS-CoV-2 (hCoV-2/human/Liverpool/REMRQ0001/2020), cultured from a nasopharyngeal swab from a patient, was passaged in Vero E6 cells [37]. The fourth virus passage (P4) was used for infections after it had been checked for deletions in the mapped reads and the stock confirmed to not contain any deletions that can occur on passage [38]. Human nCoV19 isolate/England/202012/01B (lineage B.1.1.7; Alpha variant) was from the National Infection Service at Public Health England, Porton Down, UK via the European Virus Archive (catalogue code 004V-04032). This was supported by the European Virus Archive GLOBAL (EVA-GLOBAL) project that has received funding from the European Union’s Horizon 2020 Research and Innovation Programme under grant agreement No 871029. Two Beta variants were used. For infection of K18-hACE2 mice (see below), the B.1.351 (Beta variant: 20I/501.V2.HV001) isolate [39] was received at P3 from the Centre for the AIDS Programme of Research in South Africa (CAPRISA), Durban, in Oxford in January 2021, passaged in Vero E6/TMPRSS2 cells (NIBSC reference 100978) and used here at P4. Identity was confirmed by deep sequencing at the Wellcome Trust Centre for Human Genetics, University of Oxford. For infection of BALB/c mice, a Beta variant SARS-CoV-2 isolated using transmembrane serine protease 2 (TMPRSS2)-transduced Vero E6 cells from SARS-CoV-2-infected patient nasopharyngeal samples was used [40]. The B.1.617.2 (Delta variant) hCoV-19/England/SHEF-10E8F3B/2021 (GISAID accession number EPI_ISL_1731019) was kindly provided by Prof. Wendy Barclay, Imperial College London, London, UK through the Genotype-to-Phenotype National Virology Consortium (G2P-UK). Sequencing confirmed it contained the spike protein mutations T19R, K77R, G142D, Δ156-157/R158G, A222V, L452R, T478K, D614G, P681R, D950N. The B.1.1.529/BA.1 (Omicron variant) isolate M21021166 was originally isolated by Prof Gavin Screaton, University of Oxford, UK [41] and then obtained from Prof. Wendy Barclay, Imperial College London, London, UK through G2P-UK. Sequencing confirmed it contained the spike protein mutations A67V, Δ69–70, T95I, G142D/Δ143–145, Δ211/L212I, ins214EPE, G339D, S371L, S373P, S375F, K417N, N440K, G446S, S477N, T478K, E484A, Q493R, G496S, Q498R, N501Y, Y505H, T547K, D614G, H655Y, N679K, P681H, N764K, A701V, D796Y, N856K, Q954H, N969K, L981F. The titers of all isolates were confirmed on Vero E6 cells and the sequences of all stocks confirmed.

Influenza virus A/HKx31 (X31, H3N2) was propagated in the allantoic cavity of 9-day-old embryonated chicken eggs at 35 °C. Titers were determined by an influenza plaque assay using MDCK cells [38].

Murine gammaherpesvirus (MHV) 68 clone g2.4 was used in the study and characterized as previously described [35].

### 2.2. Animals and Virus Infections

Animal work was approved by the local University of Liverpool Animal Welfare and Ethical Review Body and performed under UK Home Office Project Licence PP4715265 and approved by the Animal Experimental Board of Finland (license number ESAVI/28687/2020), respectively. It was undertaken in accordance with locally approved risk assessments and standard operating procedures. Mice carrying the human ACE2 gene under the control of the keratin 18 promoter (K18-hACE2; formally B6.Cg-Tg(K18-ACE2)2Prlmn/J) were purchased from Jackson Laboratories and Charles River, C57BL/6J mice from Charles River and BALB/c mice from Envigo. Mice were maintained under SPF barrier conditions in individually ventilated cages.

For SARS-CoV-2 infection, 6–8-week-old or 10–11-month-old (cohorts 6c and 7b) male and female K18-hACE2 mice were anaesthetized lightly with isoflurane and inoculated intranasally with 50 µL of sterile PBS containing 10^4^ PFU (Pango lineage B) or 10^2^/10^3^ PFU (Pango lineage B, Alpha, Beta, Delta, Omicron variants) of SARS-CoV-2. For SARS-CoV-2 Beta variant infection of BALB/c mice (cohort 5), 12-week-old female BALB/c mice were anaesthetized lightly with isoflurane and inoculated intranasally with 50 µL containing 2 × 10^5^ PFU of the virus.

For IAV infections (cohorts 8–10), 6–8-week-old male and female BALB/c, C57/Bl6 and K18-hACE2 mice were anaesthetized lightly with KETASET i.m. and inoculated intranasally with 10^3^ PFU of IAV X31 in 50 µL of sterile PBS.

For MHV-68 infection, 6-week-old female C57BL/6J mice were anaesthetized lightly with isoflurane and infected intranasally with 4 × 10^5^ PFU of MHV-68.

Mock-infected C57Bl/6 mice (n = 5) served as controls for the normal/constitutive expression of immunohistological markers and as additional negative controls for viral antigen staining by immunohistology.

In each experiment, animals were randomly assigned into groups. Mock-infected mice served as controls. Mice were monitored for any clinical signs and weighed.

None of the experiments was undertaken for the purpose of this study but were conducted in the course of other studies reported elsewhere [38,40,42]. Mice were dissected immediately after death and lungs collected for downstream processing. For the present study, animals were selected retrospectively from cohorts infected with the different viruses where vasculitis and perivascular infiltrates had been mentioned in the initial histopathological descriptions undertaken by one of the authors (A.Kipar). Information on mice, virus doses and the days post-infection at which animals were sacrificed by an overdose of pentabarbitone is provided in Table 1. In all animals, viral infection was confirmed by RT-PCR and immunohistology for viral nucleoprotein (NP), as previously reported [38,40].

### 2.3. Tissue Collection, Preparation and Processing

The left lungs (right lungs were subjected to qRT-PCR that determined viral loads) from all animals were collected and fixed in 10% neutral buffered formal saline for 24–48 h, then transferred to 70% ethanol until they were trimmed and routinely paraffin wax embedded.

### 2.4. Histology, Immunohistology

Consecutive sections (3–5 µm) were either stained with hematoxylin and eosin (HE) or used for immunohistochemistry (IH). IH was performed using the horseradish peroxidase (HRP) method to detect viral antigen in all examined tissues in all animals and to identify macrophages (Iba1+), T cells (CD3+), B cells (CD45R/B220+), vascular endothelial cells (CD31+), laminin, intercellular adhesion molecule 1 (ICAM-1; CD54) and vascular cell adhesion molecule (VCAM-1; CD106) in selected, representative cases of each cohort. Antibodies and detection systems are listed in Supplementary Table S1. Briefly, after deparaffination, sections underwent antigen retrieval in citrate buffer (pH 6.0) or Tris/EDTA buffer (pH 9) for 20 min at 98 °C or with Fast Enzyme for 5 min at room temperature (RT), followed by incubation with the primary antibodies (diluted in dilution buffer, Agilent Dako, Santa Clara, CA, USA). This was followed by blocking of endogenous peroxidase (peroxidase block, Agilent Dako) for 10 min at RT and incubation with the appropriate secondary antibodies/detection systems, all in an autostainer (Dako Agilent or Ventana, Oro Valley, AZ, USA). Sections were subsequently counterstained with hematoxylin.

The lungs of mock-infected control K18-hACE2, C57BL/6J and BALB/c mice served as normal lung controls for laminin, ICAM-1 and VCAM-1 and a lymph node from a normal mouse for the leukocyte markers. Sections incubated without the primary antibodies served as negative controls.

### 2.5. RNA Sequencing and Bioinformatic Analysis

Illumina RNA sequencing was undertaken on the lungs of hACE2 mice from cohorts 1 (SARS-CoV-2 Pango B infection, 3 and 7 dpi) and 9 (IAV infection, 6 dpi) as well as mock-infected control animals as previously described [38].

Trimmed paired end sequencing reads were inputted into salmon (v1.5.2) using the -l A–validateMappings–SeqBias–gcBias parameters. Quant files generated with salmon were imported into RStudio (4.1.1) using tximport to infer gene expression. The edgeR package (3.34.1) was used to normalize sequencing libraries and identify differentially expressed genes, defined as at least a 2-fold difference from the mock-infected group and a false discovery rate (FDR) less than 0.05. Differential gene expression data were used for gene ontology overrepresentation analysis of biological process terms in each group using the compareCluster function with enrichGO in the ClusterProfiler package (4.0.5) program in R. Code used to analyze data is available at https://github.com/Hiscox-lab/k18-hACE2-coinfection-transcriptomics/vasculitis. Sequencing reads are available under BioProject ID: PRJNA886870 in the Short Read Archive (SRA).

In the subsequent analysis, we focused on biological process terms that were of interest in the context of vasculitis and the recruitment of leukocytes into the tissue and on those that would indicate differences in the virus-induced pathological processes, i.e., affecting the respiratory and alveolar epithelium.

### 2.6. Morphometric Analysis

A morphometric analysis of the VCAM-1 staining of the lungs was undertaken in twenty mice (mock infected (n = 5), MHV-68 infected (5 dpi; n = 5, cohort 11), influenza virus A infected (5 dpi; n = 5, cohort 10), SARS-CoV-2 Delta infected (6/7 dpi; n = 5, cohort 6a and 6b)). The stained slides were scanned (NanoZoomer 2.0-HT; Hamamatsu, Hamamatsu City, Japan) and one longitudinal section of the lung lobe of each animal quantitatively analyzed using the Visiopharm 2022.01.3.12053 software (Visiopharm, Hoersholm, Denmark).

The morphometric analysis served to quantify the area in each lung section that stained positive for VCAM-1. For each section, the lung was manually outlined and annotated as a region of interest (ROI), manually excluding artefactually altered areas. An APP (Analysis Protocol Package), based on a threshold method, was designed in Visiopharm and run on each ROI to measure its total area (µm^2^) as well as the area of tissue showing a positive reaction (positive area; µm^2^). The percentage of positive area (%), expressed as the ratio between the positive area and the total area, was obtained for each animal in Excel (Microsoft Office 2019; Microsoft, Redmond, WA, USA), according to the following formula: ([positive area (µm^2^)]/[total area (µm^2^)]) × 100.

### 2.7. Statistical Analysis

To determine if the virus-infected animals show an increase in VCAM-1 immunostaining in the lung, the values for the percentage of positive area (%) of each of those groups (MHV-68, IAV, SARS-CoV-2) were compared to the control group (mock infected) and with each other. The statistical analyses were performed in R (R, version 4.2.2 (2022-10-31 ucrt), R Foundation for Statistical Computing, Vienna, Austria), using RStudio (RStudio: Integrated Development Environment for R, version 2022.12.0+353, Posit Software, PBC, Boston, MA, USA) with the following packages: ggplot2 (3.4.2), ggsignif (0.6.4) and MKpower (0.7).

The descriptive statistics (mean, median, range, quantile, variance, standard deviation) were determined for the infected groups and the control group, while also testing for normality of the data (Shapiro–Wilk normality test). The equality of the variances (F-test, two sided) was evaluated for all comparison pairs and these pairs were subsequently compared with either a *t*-test or a Welch test (two sided, two sample), under the null hypothesis (H0) that there was no difference in the mean percentage of positive area (%) between the different groups. The level of significance of the *t*-test and the Welch test was at 5% (95% confidence interval).

For all groups (mock infected, MHV-68, IAV, SARS-CoV-2), the result of the Shapiro–Wilk normality test indicated that the data had a normal distribution. The F-test, conducted under the null hypothesis (H0) that the variance of both groups in a comparison pair was equal, revealed that while the variances for the virus-infected groups were equal for the evaluated pairs (MHV-68–IAV; MHV-68–SARS-CoV-2; SARS-CoV-2–IAV), the variances were not equal for the virus-infected–mock-infected comparison pairs (control–MHV-68; control–IAV; control–SARS-CoV-2). Therefore, the first were analyzed with a *t*-test, while the second were analyzed with a Welch test.

## 3. Results

### 3.1. Pulmonary Vasculitis Is a Consistent Feature of SARS-CoV-2 Infection in Mouse Models

To assess whether the vascular response (vasculitis; endothelialitis) reported in the lungs of patients with COVID-19 [6,7,8,9,10,12,13] and in association with experimental SARS-CoV-2 infections [12,14,15,16,17,18,19] is also a feature in the mouse model and, if so, to determine its occurrence in the course of infection, we examined groups of K18-hACE2 mice that had been infected with SARS-CoV-2 Pango lineage B (a variant from the initial outbreak in the UK, strain hCoV-19/England/Liverpool_REMRQ0001/2020 [38], at 10^3^ and 10^4^ PFU; an Alpha variant (B.1.1.7), a Beta variant (B.1.351), a Delta variant (B1.617.2) and a near clinical (B.1.1.529) Omicron BA1 variant isolate from the UK [41] (all at 10^3^ PFU) at time points of 3 to 7 days post-infection (dpi), and, selectively, of two different age groups (6–12 weeks; 10–11 months) were used. We also examined Beta variant-infected BALB/c mice at 4 dpi. The animals included in the present investigation were all part of other studies, hence information on the course and extent of weight loss after infection and viral loads based on quantitative RT-PCRs for SARS-CoV-2N1 has been provided elsewhere [38,40,42,43]. Similarly, the principal histological changes and viral antigen expression pattern have also been reported [24,38,40]. Briefly, at day 3 post-infection (Pango lineage B, Alpha, Beta and Delta variants, K18-hACE2 mice), viral antigen expression was very widespread in the lungs, in both type I and II pneumocytes in alveoli, and associated with only occasional degenerate cells (Supplementary Figure S1A,B). In the BALB/c mice infected with the Beta variant and examined at 4 dpi, viral antigen expression was restricted to individual and small patches of positive alveoli despite infection with a high dose of 2 × 10^5^ PFU (Supplementary Figure S1C) [40]. A few days later, at 5–7 dpi (Pango lineage B, Alpha, Beta and Delta variants, K18-hACE2 mice), viral antigen expression was generally less diffuse but rather concentrated in disseminated variably sized patches of alveoli that mostly appeared unaltered. However, there was generally also evidence of acute pneumonia with pneumocyte and alveolar macrophage desquamation and often substantial macrophage-dominated infiltration; some macrophages were found to harbor viral antigen (Supplementary Figure S1D,E). For these time points, omicron BA1-infected mice were also available. A younger cohort (8–10 weeks of age, cohort 7a), examined at 6 dpi showed viral antigen expression in association with small patchy leukocyte aggregates (macrophages, neutrophils, lymphocytes) and in unaltered alveoli (Supplementary Figure S1F); this was overall less extensive than in mice infected with the other virus variants. The same applies to an older cohort, aged 10–11 months (cohort 7b), examined at 7 dpi, compared to the age-matched Delta variant-infected cohort 6c. The histological features were similar; small aggregates of macrophages with occasional neutrophils and a few degenerate cells were the main findings. Viral antigen was observed in these areas but also in small patches of unaltered alveoli.

In addition, infected animals exhibited rather consistent vascular changes at the different time points, with all virus isolates and with different inoculation doses, in both the young and older mice. 

**Figure 1:**
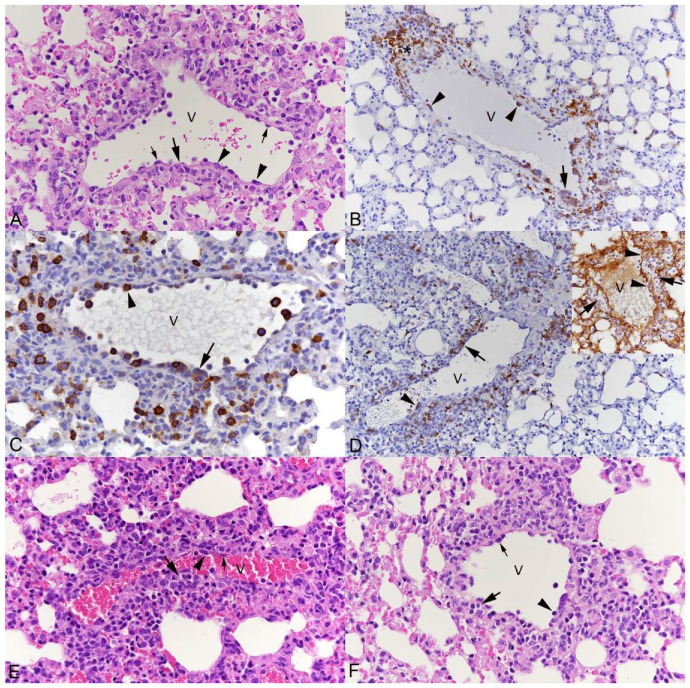
Lungs, of mice, after intranasal infection with SARS-CoV-2. Alterations of thin-walled veins (V). (**A**–**D**) K18-hACE2 mouse, infected with Pango lineage B at 10^3^ PFU, 3 dpi. (**A**) Endothelial cells appear activated, as indicated by their tombstone-like shape (small arrows). Individual leukocytes with a flattened shape are attached to the endothelium (arrowheads); leukocytes also form focal infiltrates within the vessel wall (large arrow) and surrounding the vein. HE stain. (**B**) Monocytes (Iba1+) with a flattened shape are attached to the endothelium (arrowheads); monocytes are within focal infiltrates in the vessel wall (large arrow) and surrounding the vein. The wall appears focally dissolved by the infiltrate (*). Immunohistochemistry, hematoxylin counterstain. (**C**) B cells (CD45R/B220+) are found attached to the endothelium and with a flattened shape (arrowheads) and within focal infiltrates in the vessel wall (large arrow) and surrounding the vein. Immunohistochemistry, hematoxylin counterstain. (**D**) T cells (CD3+) are found attached to the endothelium and with a flattened shape (arrowheads) and within focal infiltrates in the vessel wall (large arrow) and surrounding the vein. Inset: Staining for laminin confirms accumulation of leukocytes within the vessel wall. It indicates focal splitting of the basement membrane at the site of infiltration (arrows) and focal loss of continuity of the basement membrane at sites of leukocyte emigration (arrowheads). Immunohistochemistry, hematoxylin counterstain. (**E**) BALB/c mouse, infected with SARS-CoV-2 Beta variant at 2 × 10^5^ PFU, 4 dpi. Endothelial cells appear activated, as indicated by their tombstone-like shape (small arrow). Individual leukocytes are found attached to the endothelium and with a flattened shape (arrowhead) and as focal infiltrates within the vessel wall (large arrow) and surrounding the vein. HE stain. (**F**) K18-hACE2 mouse, 11 months, infected with SARS-CoV-2 Omicron BA.1 variant at 10^3^ PFU, 7 dpi. Endothelial cells appear activated, as indicated by their tombstone-like shape (small arrow). Individual leukocytes with a flattened shape are attached to the endothelium (arrowhead); leukocytes also form focal infiltrates within the vessel wall (large arrow) and surrounding the vein. HE stain.

The **thin-walled pulmonary veins** exhibited the typical features of leukocyte recruitment, with morphological evidence of endothelial cell activation and leukocyte rolling, emigration and perivascular accumulation, with focal loss of continuity of the vessel wall lining (Figure 1). The leukocytes involved in this process were monocytes (Iba1+), T cells (CD3+) and B cells (CD45R/B220+) in similar proportions (Figure 1B–D). The extent of their perivascular accumulation varied between individual vessels but often appeared to be most intense in proximity to areas of parenchymal infection (Supplementary Figure S1D,E).

**Figure 2:**
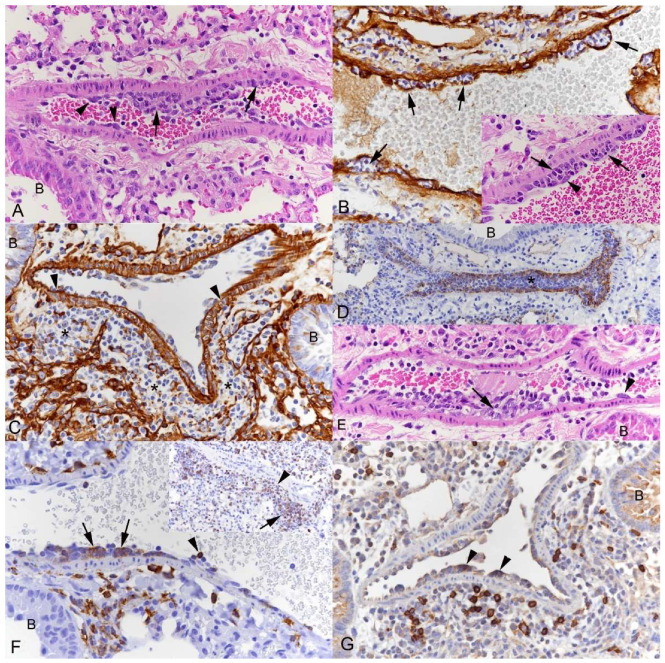
Lungs, K18-hACE2 mice after intranasal infection with SARS-CoV-2. Alterations of muscular veins. (**A**,**B**) Pango lineage B infection at 10^3^ PFU, 3 dpi. (**A**) Muscular vein with activated endothelial cells (arrowheads) and leukocyte aggregates in direct contact with the endothelium (arrows). The perivascular connective tissue appears loose, indicating perivascular edema. HE stain. (**B**) Staining for laminin shows that the leukocyte aggregates are located beneath the endothelium and indicates they sit between the basement membranes of the endothelium and the smooth muscle cells, respectively (arrows). The inset (HE stain) confirms that the leukocyte aggregates form subendothelial cushions (arrows). Arrowhead: Endothelial cell. Immunohistochemistry, hematoxylin counterstain. (**C**) Pango lineage B infection at 10^3^ PFU, 7 dpi. Staining for laminin shows subendothelial cushion formation and the presence of leukocytes in the media, between smooth muscle cells (arrowheads). There are larger leukocyte aggregates surrounding the vessel (*). Immunohistochemistry, hematoxylin counterstain. (**D**) Delta variant infection at 10^3^ PFU, 7 dpi. Leukocytes appear to completely occlude the lumen of a muscular artery (*). Staining for CD31 highlights the endothelial cell layer. B: Bronchiole. Immunohistochemistry, hematoxylin counterstain. (**E**) Omicron BA.1 variant infection at 10^3^ PFU, 10-month-old mouse, 7 dpi. Muscular artery with activated endothelial cells (arrowhead) and subendothelial leukocyte cushions (arrow). B: Bronchiole. HE stain. (**F**,**G**) Infiltrating leukocytes. (**F**) Pango lineage B infection at 10^3^ PFU, 3 dpi and 7 dpi (inset). Staining for the monocyte/macrophage marker Iba1 shows monocytes attached to the endothelium (arrowhead), forming the subendothelial leukocyte cushions (arrows) and aggregating outside the vessel. Inset: Staining for the T cell marker CD3 highlights that T cells are infiltrating the vascular wall (arrowhead) and represent a substantial proportion of the leukocytes accumulating outside the vessel. (**G**) Pango lineage B infection at 10^3^ PFU, 7 dpi. B cells (CD45R/B220+) within small subendothelial cushions (arrowheads) and among the leukocytes accumulating outside the vessel. Immunohistochemistry, hematoxylin counterstain. B: Bronchiole.

The mouse lung also has thicker-walled veins with a muscular layer composed of cardiac and smooth muscle cells and a diameter of 70–250 µm (from now on called “muscular veins”; Figure 2A); these are found in proximity to bronchioles [44]. In all examined groups of mice, the inflammatory changes also involved **muscular veins** (Figure 2). Endothelial cells appeared activated, with clusters of leukocytes attached to them and forming subendothelial clusters that were located between the basement membranes of endothelial and smooth muscle cells, as indicated by staining for laminin (Figure 2A,B). Leukocytes were also seen between smooth muscle cells of the media and were found accumulating just outside the latter (Figure 2C). They were extremely abundant in some vessels where they appeared to entirely fill the lumen (Figure 2D), consistent with massive leukocyte recruitment into the tissue. Despite the overall less severe inflammatory processes, the same processes were also observed in muscular veins in Omicron-infected mice (Figure 2E). The inflammatory processes comprised monocytes and T and B cells; monocytes dominated in the subendothelial cushions, although B cells were also seen beneath the endothelium, and all three types were found within and accumulating around the vessel walls (Figure 2F,G).

**Figure 3:**
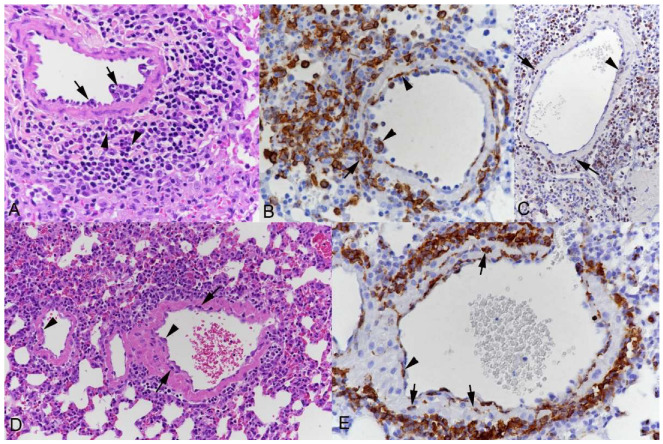
Lungs of mice after intranasal infection with SARS-CoV-2. Alterations of muscular arteries. (**A**,**B**) K18-hACE2 mouse, infected with Pango lineage B at 10^3^ PFU, 7 dpi. (**A**) Muscular artery with some subendothelial leukocyte aggregates (arrows) and perivascular infiltration, containing mononuclear cells and some neutrophils (arrowheads). HE stain. (**B**) Staining for Iba1 shows monocytes in the subendothelial leukocyte aggregates, within the media (partly with elongated shape) and surrounding the vessel wall. Immunohistochemistry, hematoxylin counterstain. (**C**) K18-hACE2 mouse, infected with Delta variant at 10^3^ PFU, 7 dpi. Staining for CD3 shows T cells in subendothelial (arrowhead) and deeper layers (arrows) of the media as well as in the perivascular infiltrate. Immunohistochemistry, hematoxylin counterstain. (**D**,**E**) BALB/c mouse, infected with Beta variant at 2 × 10^5^ PFU, 4 dpi. (**D**) Arteries with activated endothelial cells (arrowheads), scattered leukocytes within the media (arrow) and mild perivascular leukocyte infiltration. HE stain. (**E**) Staining for Iba1 highlights monocytes beneath the endothelium (arrowhead), within the media (arrows) and abundant in the perivascular infiltrate. Immunohistochemistry, hematoxylin counterstain.

**Pulmonary muscular arteries**, which can in mice be as small as 20 µm in diameter [44], were also found to be affected in all groups of mice (Figure 3). They exhibited activated endothelial cells, occasional subendothelial leukocyte cushions and leukocytes (mainly represented by monocytes) in the vascular wall, and focal to circular periarterial leukocyte infiltrates. The latter comprised macrophages and T and B cells and often a few neutrophils (Figure 3).

### 3.2. Respiratory Virus Infections, with Variable Degree of Alveolar Damage, and with or without Damage to Respiratory Epithelium, Elicit a Stereotypic Vascular Response

In order to determine whether the described vascular response is specific for SARS-CoV-2 infections or also a feature of other respiratory viral infections in the murine model, we included cohorts of mice that were infected with IAV and MHV-68 and examined them at time points with most extensive pulmonary damage (IAV: 2, 5, 6, 7 dpi; MHV-68: 5 dpi) (Supplementary Figure S2).

**Figure 4:**
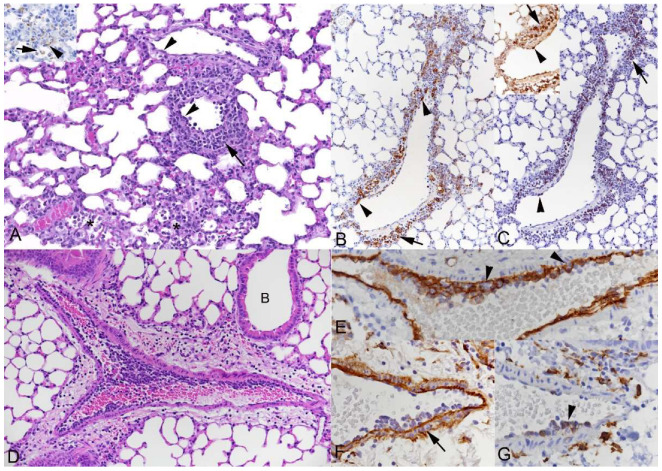
Lungs of C57BL/6J mice after intranasal infection with IAV X31 at 10^3^ PFU. (**A**–**D**) C57/Bl6 mouse, 5 dpi. Leukocyte recruitment from veins in the parenchyma and perivascular accumulation (arrow). (**A**) Focal parenchymal necrosis and loss of infected alveolar epithelial cells, with mild leukocyte infiltration (*); inset: Viral antigen expression in type I pneumocytes (arrowhead) and degenerate, desquamated cells (arrow). Veins in the vicinity exhibit activated endothelial cells (arrowheads) and perivascular leukocyte infiltration (arrow). HE stain. (**B**,**D**) K18-hACE2 mouse, 6 dpi. Affected veins. Monocytes (Iba1+; B), T cells (CD3+; C) and to a lesser extent B cells (CD45R/B220+; C: Inset) are recruited from the blood and seen attached to the endothelium (arrowheads) and accumulating outside the vein (arrows). Immunohistochemistry, hematoxylin counterstain. (**D**–**G**) Muscular vein with leukocytes attached to the endothelium and small subendothelial leukocyte aggregates. (**D**) B: Bronchiole. HE stain. (**E**,**F**) Staining for CD31 (**E**), an endothelial cell marker, highlights leukocytes beneath the endothelium (arrowheads), and staining for laminin (**F**) shows their presence between the basement membranes of endothelial cells and smooth muscle cells (arrow). Immunohistochemistry, hematoxylin counterstain. (**G**) Monocytes (Iba1+) are present attached to and beneath the endothelial cells (arrowhead). Immunohistochemistry, hematoxylin counterstain.

As described [36,38], intranasal infection of mice with the non-lethal IAV X31 strain leads to extensive necrosis of the airway epithelium as a direct effect of viral infection; the virus spreads from the distal airway into the alveoli where it also induces necrosis and raises an inflammatory response (Supplementary Figure S2A,B), with involvement of blood vessels in proximity to affected bronchioles and parenchyma. The thin-walled veins exhibited changes consistent with leukocyte recruitment and perivascular accumulation of monocytes/macrophages (Iba1+), T cells (CD3+) and B cells (CD45R/B220+) (Figure 4A–C). Muscular veins showed activated endothelial cells, attachment of clusters of leukocytes and leukocytes between the basement membranes of endothelium and smooth muscle cells (Figure 4D–G). Changes in muscular arteries did generally not go beyond activation of endothelial cells.

**Figure 5:**
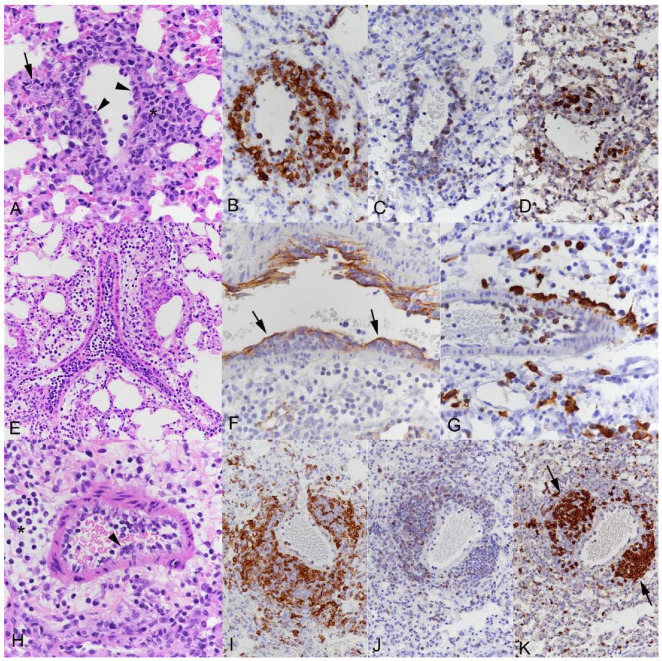
Lungs of C57BL/6J mice after intranasal infection with MHV-68 at 4 × 10^5^ PFU. (**A**–**D**) Thin-walled vein. (**A**) Leukocyte attachment to endothelial cells (arrowheads) and perivascular accumulation (*). Arrow: Small focal, virus-induced parenchymal lesion. HE stain. (**B**–**D**) Monocytes/macrophages (Iba1+; B), T cells (CD3+; C) and B cells (CD45R/B220+; D) are all recruited into the infiltrates. Immunohistochemistry, hematoxylin counterstain. (**E**–**G**) Muscular vein. (**E**) Abundant leukocytes are present in the lumen and attached to the endothelium. HE stain. (**F**) Staining for CD31, an endothelial cell marker, highlights the presence of leukocyte clusters beneath the endothelium (arrows). Immunohistochemistry, hematoxylin counterstain. (**G**) Monocytes (Iba1+) are seen within the lumen of the vein, attached to the endothelium and outside the vessel. Immunohistochemistry, hematoxylin counterstain. (**H**–**K**) Muscular artery. (**H**) Endothelial cells are activated, with leukocytes attached to the endothelium (arrowhead). There is moderate periarterial edema (*). HE stain. (**I**–**K**) Monocytes/macrophages (Iba1+; **I**), T cells (CD3+; **J**) and B cells (CD45R/B220+; **K**) are all recruited into the infiltrates; B cells form small follicle-like aggregates (arrows). Immunohistochemistry, hematoxylin counterstain.

With MHV-68 infection, despite its more subtle parenchymal damage, with only minimal alveolar damage and parenchymal as well as peribronchial and perivascular infiltrates (Supplementary Figure S2), inflammatory changes affecting all types of vessels were again observed (Figure 5) and generally without evidence of a spatial relation to virus-induced alveolar changes. Both veins and arteries were affected, with distinct recruitment of monocytes (Iba1+), T cells (CD3+) and B cells (CD45R/B220+) into the perivascular space (Figure 5B–D,I–K); occasionally, B cells were found to form small follicle-like aggregates, as previously described [35]. Again, there were muscular veins packed with leukocytes and subendothelial leukocyte cushions (Figure 5E,F). The latter comprised mainly monocytes (Iba1+; Figure 5G).

Taken together, these results indicate that viral infection of the lung targeting the respiratory and/or alveolar epithelium elicits a rather stereotypic inflammatory response mediated by the vasculature and a broad range of leukocytes. Interestingly, a previous study using the bleomycin model of acute lung injury and repair reported a very similar histological picture, with microscopic changes indicative of leukocyte migration across the wall of pulmonary arteries and veins, and their accumulation in the perivascular interstitium [45].

### 3.3. The Transcriptomic Analysis Reflects the Differences in Viral Pathogenicity but Also Confirms the Stereotypic Vascular Response in SARS-CoV-2 and IAV Infection

**Figure 6:**
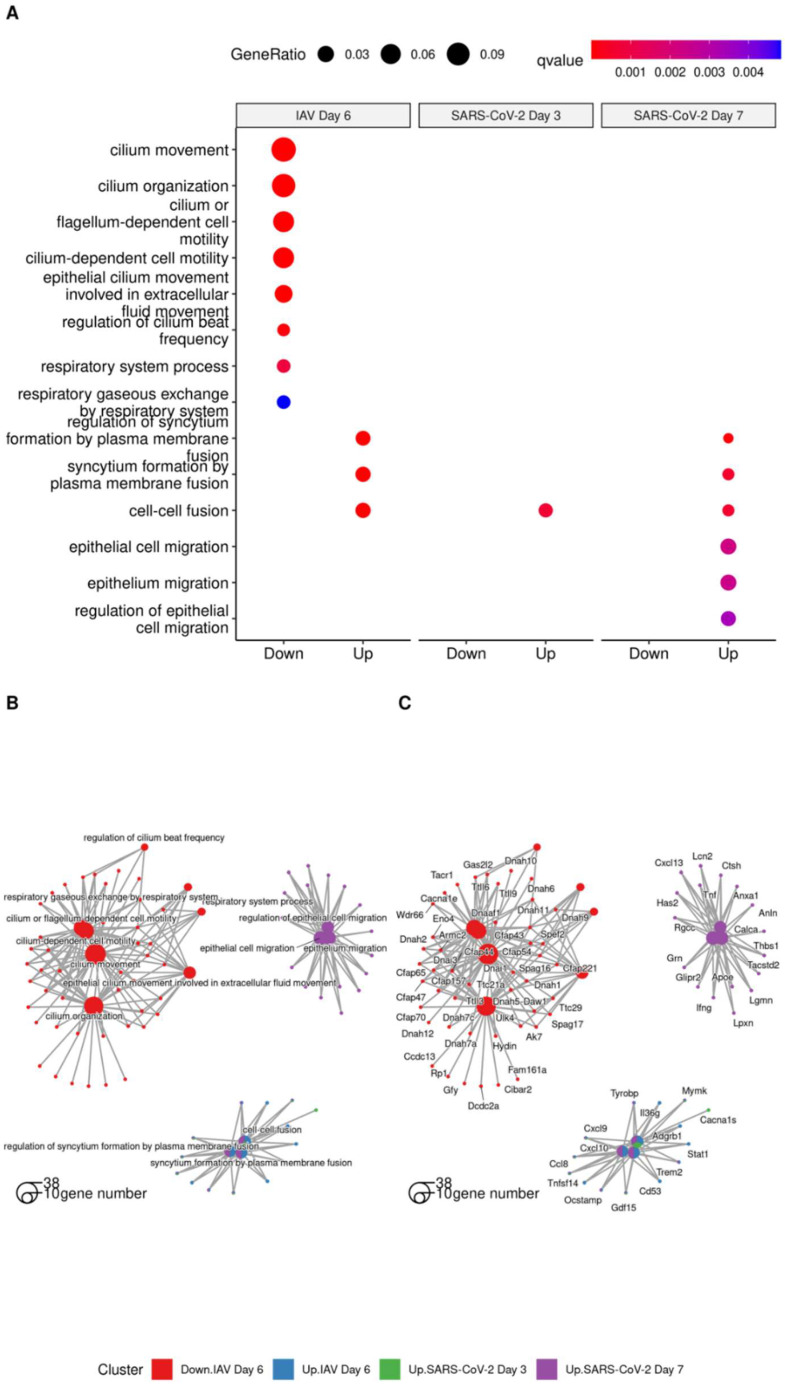
(**A**) GO terms associated with “epithelial alterations” following analysis of differentially expressed genes using clusterProfiler. Cnetplot was used to show genes associated with ontology terms highlighting biological complexity as genes belong to multiple biological process annotations. (**B**) shows the category annotation, (**C**) shows the gene annotation.

To further explore the vascular response and to investigate the difference with regard to the damage that SARS-CoV-2 and IAV induce in the lung at the transcriptional level, we utilized RNAseq data generated previously [38]. Differential gene expression analysis was conducted, followed by an enrichment analysis with ClusterProfiler. Overrepresented biological process terms associated with epithelial alterations and vasculitis were extracted for visualization and to identify genes that were associated with enrichments. Results from compareCluster were plotted with the cnet function to examine the networks within the dataset (Supplementary Figure S3). As we were interested in specific transcriptional responses, we interrogated the data for specific terms. Figure 6A shows selected gene ontology terms associated with epithelial damage. At day 6 post-IAV infection, downregulation of transcripts associated with cilium movement and organization was observed, consistent with the respiratory epithelial damage histologically observed at this time point [36,38]. Figure 6B,C reveal the biological complexity, where the same gene contributes to multiple annotations.

In contrast, at day 3 post-SARS-CoV-2 Pango B infection, the only evidence of epithelial damage is upregulation of cell–cell fusion transcripts, which would be consistent with the observed syncytial cell formation [38]. At day 7 of SARS-CoV-2 infection, however, there was an upregulation of epithelial cell migration transcripts, which clusters separately from IAV cilial damage terms and cell fusion terms which were shared across all groups. This would be consistent with onset of attempts at epithelial regeneration, as is observed in alveoli at this stage [38]. Results from differential gene expression and the enrichment analysis outputs are supplied as supplementary material (Supplementary Data S1 and S2).

**Figure 7:**
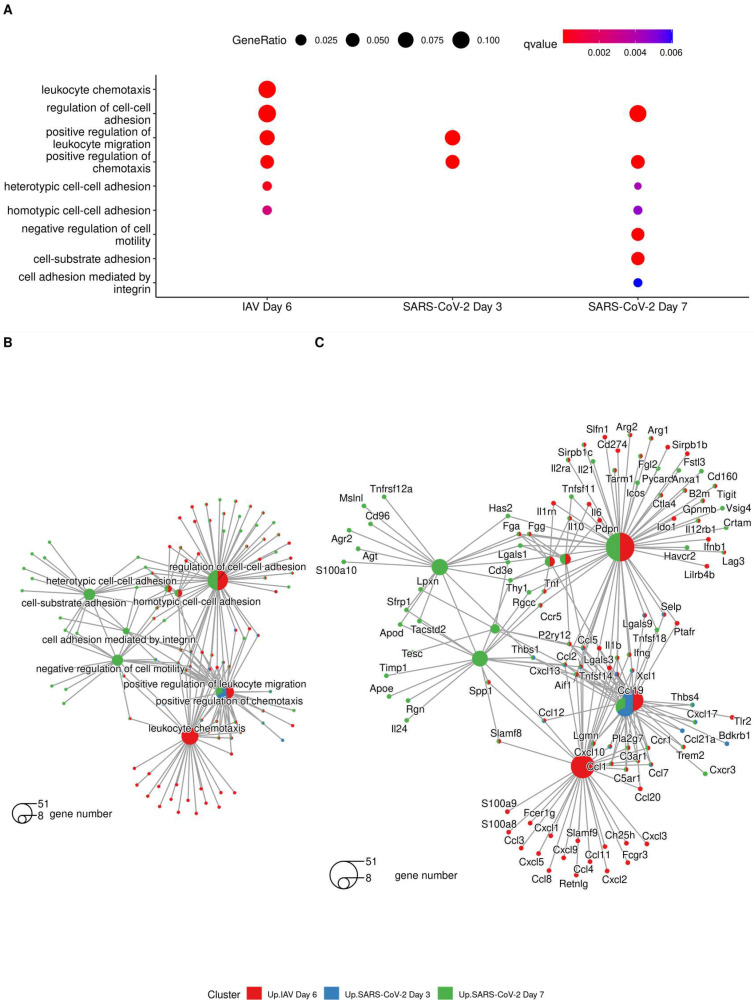
(**A**) GO terms associated with “vasculitis” following analysis of differentially expressed genes using clusterProfiler. Cnetplot was used to show genes associated with ontology terms highlighting biological complexity as genes belong to multiple biological process annotations. (**B**) shows the category annotation, (**C**) shows the gene annotation.

Similarly, we assessed terms that were associated with the inflammatory processes associated with the virus infections. The search for gene ontology terms yielded a long list of upregulated transcripts. Most of these are consistent with an acute viral infection and an innate immune response, as reported before [38]. Also included are transcripts that directly relate to the observed vascular processes (Figure 7). Several transcripts related to leukocyte migration and chemotaxis were upregulated with both viruses, others only with IAV infection and/or SARS-CoV-2 Pango B infection at one or both time points. Overall, these results support the other findings, i.e., that despite the different viral pathogenesis, both viruses induce a similar inflammatory response.

### 3.4. The Vascular Response after Respiratory Virus Infections Is Associated with Increased Expression of Adhesion Molecules in the Lungs

**Figure 8:**
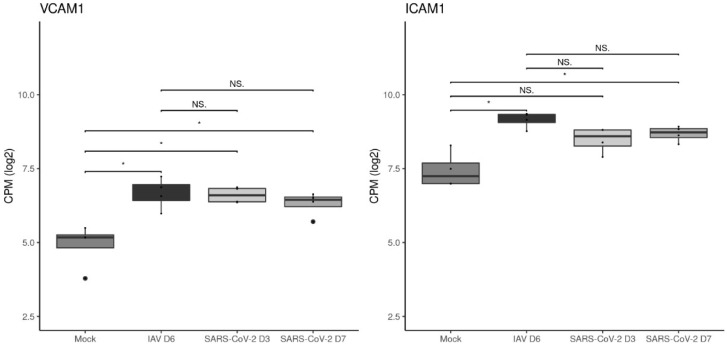
The TMM normalized log2 counts per million (cpm) of VCAM-1 and ICAM-1 for each animal within each group (n = 4) were plotted as boxplots. Pairwise comparisons between mock-infected and virus-infected groups and between IAV- and SARS-CoV-2 Pango B-infected groups were made with the “ggsignif” package and a Wilcoxon test. * *p* < 0.05, NS: Not significant. The points (●) represent outliers.

The previous study reporting on the vascular changes in the bleomycin mouse model of acute injury and repair provided evidence that the adhesion molecules VCAM-1 and ICAM-1 mediate leukocyte recruitment via pulmonary arteries and veins into the lungs [45]. This prompted us to investigate the expression of VCAM-1 and ICAM-1 at both the RNA and protein level. Gene expressions of VCAM-1 and ICAM-1 were significantly higher in infection groups compared to mock-infected animals, with the exception of ICAM-1 when comparing mock- and SARS-CoV-2-infected animals at day 3. When comparing between infection groups, no significant differences were identified, however, there was a trend of higher ICAM-1 transcription in IAV-infected animals (Figure 8).

Immunohistological staining for both adhesion molecules was then performed not only on the lungs of SARS-CoV-2 Delta-infected K18-hACE2 mice (cohorts 6a, b; n = 5; 6 dpi) and IAV-infected C57BL/6J mice (cohort 10; n = 5; 5 dpi) but also MHV-68-infected C57BL/6J mice (cohort 11; n = 5; 5 dpi), due to the similarity of the vascular and perivascular changes. A group of five mock-infected age-matched C57BL/6J mice which we confirmed to show the same VCAM-1 and ICAM-1 expression pattern as K18-hACE2 mice served as controls.

**Figure 9:**
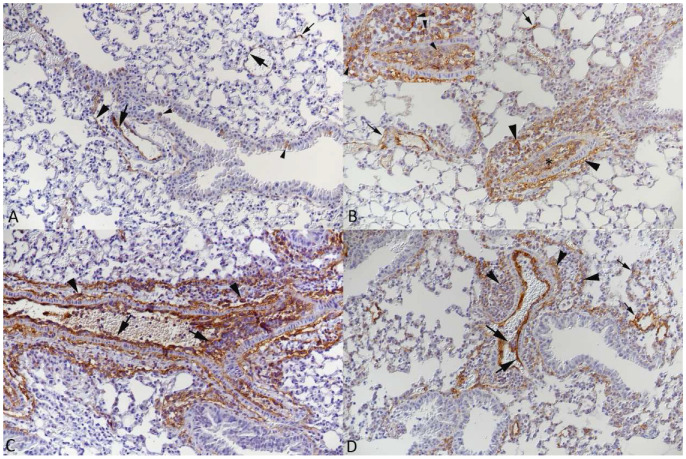
Lungs of mice. VCAM-1 expression. (**A**) Mock-infected C57BL/6J mouse. VCAM-1 expression is most prominent in endothelial cells in larger vessels such as (muscular) veins (large arrows) and to a lesser extent in occasional capillaries of alveolar walls (small arrow). A cell-free reaction is seen in the connective tissue surrounding the larger vessels (large arrowhead). Occasional respiratory epithelial cells in bronchioles show a weak reaction (small arrowhead). (**B**) K18-hACE2 mouse infected with 10^2^ PFU of SARS-CoV-2 Delta, 6 dpi. VCAM-1 expression shows a similar pattern. However, there is stronger perivascular staining, in the connective tissue and on infiltrating leukocytes (arrowheads, also in inset), and more widespread expression in capillary endothelial cells in alveolar walls (small arrows). Strong expression is also seen in association with the abundant recruited leukocytes in the lumen of an affected muscular vein (*); inset: Arrowhead. (**C**) C57BL/6J mouse infected with 10^3^ PFU of IAV X31, 5 dpi. Closer view of a muscular vein with strong VCAM-1 expression in endothelial cells, also covering subendothelial leukocyte aggregates (arrows), and outside the vessel, in the perivascular connective tissue and on infiltrating leukocytes (arrowheads). (**D**) C57BL/6J mouse infected with 4 × 10^5^ PFU of MHV-68, 5 dpi. Strong VCAM-1 expression in endothelial cells of an affected muscular vein, also covering subendothelial leukocyte aggregates (large arrows) as well as in the perivascular connective tissue and, less intensely, on infiltrating leukocytes (arrowheads). There is also relatively widespread staining of capillary endothelial cells in alveolar walls (small arrows). Immunohistochemistry, hematoxylin counterstain.

In the uninfected control lung parenchyma, VCAM-1 expression was observed in endothelial cells of blood vessels and, rarely, capillaries in the alveolar walls. There was some cell-free, possibly connective tissue-associated, reaction around vessels. Limited weak to moderate staining was also seen in respiratory epithelial cells (Figure 9A). The expression pattern did not change with viral infection and the presence of the vascular changes. However, there was evidence of more extensive expression in endothelial cells and a slightly more widespread expression in capillary endothelial cells in alveolar walls. In affected vessels, both rolling/recruited and perivascular leukocytes as well as the perivascular connective tissue also showed some VCAM-1 expression (Figure 9B–D).

**Figure 10:**
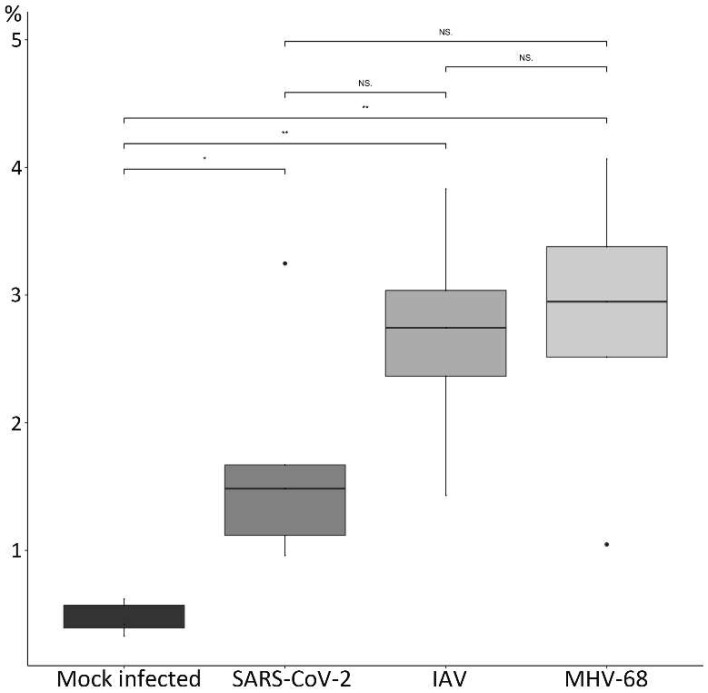
Lungs of mice. Morphometric analysis of VCAM-1 expression in the lungs of mock-infected mice and mice infected with SARS-CoV-2 Delta variant, IAV X31 and MHV-68 and examined between 5 and 7 dpi. Box and whisker plots showing the percentage of positive area (%) for the different groups. The three virus-infected groups show a significant increase in the expression of VCAM-1 compared to the control group. The significance levels for the mock-infected–virus-infected comparison pairs were calculated with a Welch test, while the significance levels for the comparisons between virus-infected groups were calculated with a *t*-test. NS: Not significant, ** = 0.01, * = 0.05. The points (●) represent outliers.

Morphometric analysis then served to determine quantitative differences in the overall expression of VCAM-1 after virus infection. This confirmed a significantly higher expression in virus-infected lungs as compared to control lungs (Figure 10); the comparison between the three infected groups showed a trend with higher expression of VCAM-1 in MHV-68- and IAV-infected groups, although the pair comparisons yielded no significant differences between the virus-infected groups. The power analysis of the statistical tests yielded a power above 0.80 for only two tests (control–IAV and control–MHV-68). The detailed results of the analysis are provided in Supplementary Table S2. 

**Figure 11:**
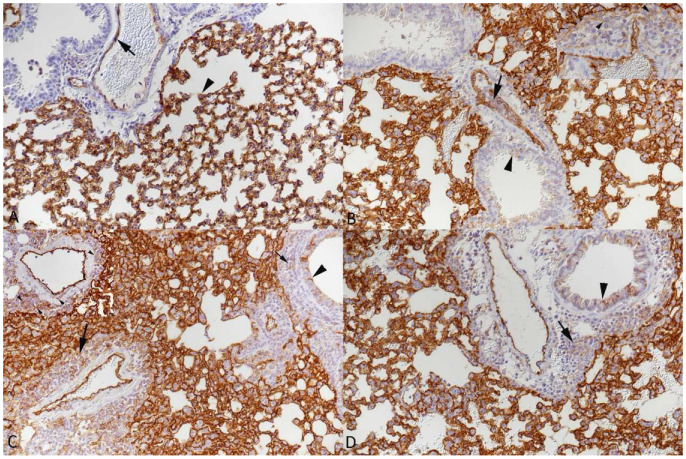
Lungs of mice. ICAM-1 expression. (**A**) Mock-infected C57BL/6J mouse. ICAM-1 is strongly expressed by endothelial cells in both larger vessels (arrow; muscular vein) and capillaries in alveolar walls (arrowhead). (**B**) K18-hACE2 mouse infected with 10^2^ PFU of SARS-CoV-2 Delta, 6/7 dpi. The ICAM-1 expression is in general identical to that observed in control mice. Endothelial cell staining highlights the interaction with attaching leukocytes (arrow), and there is weak expression also by infiltrating leukocytes in the perivascular space (inset: Arrowheads). Individual respiratory epithelial cells in bronchi exhibit a weak expression at the cell surface (arrowhead). (**C**) C57BL/6J mouse infected with 10^3^ PFU of IAV X31, 5 dpi. In bronchioles, there is consistent ICAM-1 expression by respiratory epithelial cells (arrowhead) and, weakly, in infiltrating leukocytes (small arrow). Leukocytes in the perivascular infiltrate appear positive (arrow). Inset: ICAM-1 is also expressed on the surface of emigrating and perivascular leukocytes (arrowheads). (**D**) C57BL/6J mouse infected with 4 × 10^5^ PFU of MHV-68, 5 dpi. ICAM-1 expression is very similar to that seen in the other two virus infections, with expression also by infiltrating leukocytes (arrows) and bronchiolar epithelial cells (arrowhead). Immunohistochemistry, hematoxylin counterstain.

ICAM-1 expression was overall more widespread, as was observed in endothelial cells of all blood vessels and capillaries in the alveolar walls (Figure 11A). The staining pattern remained the same after virus infection. In addition, infiltrating leukocytes in affected vessels were found to be positive, and some reaction was seen on the luminal surface of bronchiolar epithelial cells, which was least intense with SARS-CoV-2 infection (Figure 11B–D).

Due to the obviously very limited differences in expression of the adhesion molecule, quantitative analysis by morphometry was not undertaken for ICAM-1.

## 4. Discussion

The present study has employed mouse models of respiratory virus infections to challenge the concept that pulmonary vasculitis and specifically endothelialitis are phenomena unique to SARS-CoV-2 infection. For this purpose, we examined the lungs of K18-hACE2 mice and wild type mice of different ages and both sexes infected with various SARS-CoV-2 isolates at different virus doses and time points and mice infected with a non-lethal strain of IAV and a murine gammaherpesvirus (MHV-68) by histology and immunohistology, combined with an RNAseq analysis.

Regardless of the infection model, we observed a common pattern of vascular reaction. This involves thin-walled pulmonary veins, muscular veins (referring to veins with a diameter of 70–250 µm and a muscular layer composed of cardiac and smooth muscle cells [44]) and pulmonary muscular arteries and is characterized by the presence of leukocytes (monocytes, T and B cells) in the vascular lumen, attached to or beneath the endothelial lining, in the vascular wall and in the perivascular space. Although the leukocytes were observed at different levels in the vascular wall, the wall itself exhibited only minor changes; besides the general activation of endothelial cells, focal “loosening” of the wall structures was observed in association with emigration of the leukocytes. With none of the viruses, and regardless of mouse strain, infectious dose and time point of infection, was there any evidence of viral infection of endothelial cells, as investigated by immunohistology for the different viral antigens.

The results of the present study provide strong evidence that the vascular processes described in the lungs of animals infected with SARS-CoV-2 are not specific for the virus but are instead a key component of a stereotypic pulmonary response to respiratory virus infections that affect the alveolar and/or bronchiolar epithelium. This interpretation is supported by the results of our transcriptomics analyses. We have previously shown that acute infections of K18-hACE2 mice with SARS-CoV-2 Pango B and IAV lead to a similar, substantial innate immune response [38]. When we focused on the pathogenic aspects, i.e., the strong epithelial damage, we found a clear difference between the two models, as transcriptional changes consistent with the respiratory epithelial damage were only apparent with IAV infection. Data derived from epithelial cell models have reported shared gene expression responses to respiratory viruses including murine hepatitis virus-1, IAV PR8 and rhinovirus RV1B [46]. Also, in human studies, H1N1 IAV was shown to cause disruption of the epithelial layer, whereas SARS-CoV-2 did not [47]. Investigating transcripts related to leukocyte migration and chemotaxis, main components of vasculitis and leukocyte recruitment into the tissue, we gathered more evidence that both viruses induce a similar inflammatory response.

It is not unlikely that the vascular changes in the lungs of human COVID-19 patients represent a similarly stereotypic response, in particular since comparable changes have also been reported with RSV infection [30]. Indeed, the vascular changes might not even be specific for virus infections but may rather represent a response to bronchiolar and alveolar epithelial damage in general [45].

The vascular processes principally recapitulate all the steps of acute inflammation, from leukocyte rolling and attachment over emigration to their accumulation outside the vessels (reviewed in [48,49]), though with a few peculiarities: (1) Monocytes are quantitatively the dominant cell population, but T and B cells are obviously recruited in parallel, alongside a few neutrophils, suggesting that the activated endothelial cells non-selectively mediate attachment of the various leukocytes simultaneously rather than sequentially. While several molecules are known to play a role in the emigration of leukocytes (ICAM-1, VCAM-1, JAM-A, JAM-C, endothelial cell-selective adhesion molecule, PECAM, CD99, CD99L2) (reviewed in [50]), the contribution of adhesion molecules such as selectins and integrins to the recruitment of leukocytes in the lung is still debated (reviewed in [51]). (2) Different from the “classical” acute inflammation, the processes are not taking place in post-capillary venules which would generally allow easiest emigration due to their thin walls. Instead, the thin-walled veins as well as thicker-walled vessels, with distinct media comprising muscle cell layers, i.e., both the muscular veins specific to the murine lungs [44] and arteries, are most likely migrated through by the emigrating leukocytes. The latter require them to pass the basement membrane and the otherwise tightly arranged muscle. Indeed, staining for laminin, a protein of the basement membrane of endothelial cells and vascular smooth muscle cells (reviewed in [52,53]), appeared rearranged with emigration, providing further evidence of leukocyte-permissive regions in the basement membrane/vascular wall (reviewed in [48]). (3) Emigrating leukocytes also form focal, cushion-like or even circular subendothelial aggregates. Without convincing morphological evidence that the leukocytes target the endothelium or, potentially, the media, this suggests a somehow stepwise emigration process. Interestingly, this phenomenon has also been observed in the acute pulmonary response to bleomycin in a mouse model where it was interpreted as an alternative mode of leukocyte influx into the lung [45]. At the same time, the presence of abundant leukocytes that appear to almost occlude the lumen of some muscular veins and arteries suggests massive activation of both endothelial cells and circulating leukocytes. The strong VCAM-1 and ICAM-1 expression of the endothelial cells and their tombstone-like shape are further indicators of their activation. Finally, there are also quite substantial perivascular leukocyte accumulations, raising the question whether the leukocytes really migrate within the tissue further away from the vessels.

We undertook an investigation into the extent of transcription and the protein expression of the adhesion molecules VCAM-1 and ICAM-1. In mice, both VCAM-1 and ICAM-1 are expressed by endothelial cells and some leukocytes, and ICAM-1 expression has also been reported in epithelial cells (including the alveolar and bronchial epithelium) and fibroblasts [54,55,56]. Upregulation of both molecules in pulmonary endothelial cells has been suspected to play a role in the recruitment of leukocytes during IAV infection (reviewed by [57]) and has been shown in both in vivo and in vitro models of SARS-CoV-2 infection [19,58]. The transcriptomics analysis revealed their significant increase in the lungs after IAV and SARS-CoV-2 (at day 7) infection. This was confirmed at the protein level by morphometry for VCAM-1 where immunohistology suggests that this is due to its upregulation in endothelial cells and expression by the recruited leukocytes. For ICAM-1, which is diffusely expressed by the capillary endothelium in alveolar walls, no such obvious difference was noted although the recruited leukocytes were also found to express the marker. Nonetheless, the results support the assumption that VCAM-1 and ICAM-1 are involved in the recruitment of leukocytes into the lungs, with both adhesion molecules being expressed in endothelial cells and emigrating leukocytes [55,56]. Supporting this hypothesis is the work undertaken in a mouse model of bleomycin-induced injury and repair which, similar to the present study, showed expression of ICAM-1 and VCAM-1 at sites of interaction between endothelium and leukocytes [45].

Interestingly, in hamsters infected with SARS-CoV-2, leukocyte infiltration of the wall of pulmonary arteries and veins has been described, suggesting that leukocyte emigration also takes place at this level in this species [17]. Taken together, there is now substantial evidence that the pulmonary veins and arteries are the main sites of leukocyte emigration in the lungs. This contradicts previous statements that the latter occurs in the capillaries [59,60] and makes perfect sense since in the lungs the diameter of a large portion of the capillaries is smaller than the diameter of a neutrophil which makes emigrating a major effort for leukocytes; indeed, neutrophil tethering and rolling can only take place in larger-diameter arterioles and venules in the lungs (reviewed in [61]).

Provided that the changes in the vascular walls are only a bystander of leukocyte emigration, the question arises as to whether the processes indeed represent a vasculitis. Vasculitis is in the broader sense defined as an inflammation of the blood vessels, with various causes [62], and hence does not necessarily require the vessel to be the target of the inflammatory processes. This is different when the term “endothelialitis/endotheliitis” is applied, as has recently been the case for the vascular changes in human COVID-19 cases and animal models of COVID-19 [6,7,18]. The articles using this term applied variable combinations of histologic features for its definition, such as an infiltration of the intima/endothelial layer by leukocytes [14,15,18] or an infiltration of the endothelial layer in association with cell death/degeneration [16,20,22]. Some authors also associated thickening of the intima and leukocytic infiltration [12] or proliferative changes of the intima and leukocyte adherence [19] with it. A PubMed search for the terms “endotheliitis” and “endothelialitis” showed a first use in 1958 when they referred to retinal lesions [63]. Since the 1980s, they were also applied to vascular changes in the kidney [64] and liver; the latter are described as “attachment of lymphoid cells to the endothelium of central or portal veins” [65]. In a recent review on vascular endothelialitis in infectious diseases, the rather vague definition “inflammation of the endothelium lining the lumen of blood vessels in association with a direct consequence of infectious pathogen invasion and the host immune response” was suggested [66]. However, the authors also mentioned the use of the term for a virus-associated inflammation of the corneal endothelium (which is not an endothelium as in blood vessels!) [66]; the term is still in use in this context (reviewed in [67]). It remains to be debated whether such versatile use of the term is helpful.

While the present study provides some mechanistic insight into the processes leading to vascular changes in respiratory virus infections, it does not identify the initial trigger; however, it provides strong evidence that infection/damage of respiratory and/or alveolar epithelium is at the core, directing further studies in this field. 

Fundamental understanding of the disease biology is of critical importance in its own right but also specifically important to drive development and rationalization of therapeutics for use for COVID-19 and other pulmonary virus threats. For COVID-19 therapeutics, a greater understanding of the anatomical and cellular target sites will enable better assessment of tissue penetration and facilitate quantitative pharmacology approaches to define exposure targets. This knowledge will help unmask new therapeutic modalities but will also enable better dose prediction for clinical development and help rationalize continued use of medicines against the backdrop of a shifting landscape of variants.

## 5. Conclusions

The present study showed that respiratory virus infections (SARS-CoV-2, IAV, MHV-68) of mice initiate a stereotypic pulmonary vascular response, mediated by arteries and veins, without endothelial infection. This stereotypic reaction is characterized by the recruitment of monocytes and T and B cells, their emigration with accumulation in the vascular wall and their perivascular location. Adhesion molecules such as VCAM-1 and ICAM-1 appear to play a relevant role in this rather non-selective process.

## Figures and Tables

**Table 1:** Study cohorts. Animal numbers differ, as mice originated from several experiments.

Cohort (Mice)	Age	Virus	PFU	DPI	No of Animals
1 (hACE2)	6–8 weeks	Pango B	10^4^	3, 7	8, 8
2 (hACE2)	6–8 weeks	Pango B	10^3^	6	6
3 (hACE2)	6–8 weeks	Alpha	10^3^	3, 6/7	4, 4
4 (hACE2)	6–8 weeks	Beta	10^3^	3, 6/7	4, 4
5 (BALB/c)	12 weeks	Beta	2 × 10^5^	4	3
6a (hACE2)	6–8 weeks	Delta	10^3^	3, 5/6, 7	4, 4, 5
6b (hACE2)	6–8 weeks	Delta	10^2^	6/7	8
6c (hACE2)	10–11 months	Delta	10^3^	7	6
7a (hACE2)	8–10 weeks	Omicron BA.1	10^3^	6	6
7b (hACE2)	10–11 months	Omicron BA.1	10^3^	7	6
8 (BALB/c)	6–8 weeks	IAV X31	10^3^	2	4
9 (hACE2)	6–8 weeks	IAV X31	10^3^	6	3
10 (C57BL/6J)	6–8 weeks	IAV X31	10^3^	5, 7	5, 5
11 (C57BL/6J)	6 weeks	MHV68	4 × 10^5^	5	5

Abbreviations: DPI—days post-infection (day of euthanasia); hACE2—K18-hACE2 mice; PFU—plaque-forming units.

## Data Availability

The data presented in this study are available in the article itself and in the supplementary material.

## Supplementary Materials

The following are available online at https://www.mdpi.com/article/10.3390/v15081637/s1, Table S1: Antibodies and detection methods used for immunohistochemistry. Table S2: Detailed results of the statistical analysis following the morphometric analysis of VCAM-1 expression in the lungs. Figure S1: Viral antigen expression in the lungs of mice after SARS-CoV-2 infection. Figure S2: Histological changes and viral antigen expression in the lungs of mice infected with IAV and MHV-68. Figure S3: The top biological process terms within the dataset before extracting biological process terms associated with epithelial involvement and vasculitis. Supplementary Data S1: Differential gene expression analysis results, including logFC of each gene and the FDR. Supplementary Data S2: Biological process results from compareCluster with clusterProfiler.
