# Supplementary material for: The Stereotypic Response of the Pulmonary Vasculature to Respiratory Viral Infections: Findings in Mouse Models of SARS-CoV-2, Influenza A and Gammaherpesvirus Infections
Source: Viruses. 2023 Jul 27;15(8):1637. doi: 10.3390/v15081637 (PMC10458810; doi:10.3390/v15081637)
Supplement: Supplementary file 1 [file viruses-15-01637-s001.zip › Supplementary Material.pdf]

Supplemental Material

Supplemental Table S1. Antibodies and detection methods used for immunohistochemistry

**Antigen**	**Antibody (clone)**	**Antigen retrieval**	**Antibody dilution, detection method**
SARS-CoV NP	pAB rabbit[a]	CB pH 6.0, 98 °C, 20 min	1:6,000, ON, 4 °C EnVision+ (HRP, Rb)[i]
IAV	goat anti-IAV (H1N1; virions)[b]	TE pH 9.0, 98 °C, 20 min	1:200, 60 min, RT Rb anti-goat Ig/HRP[f]
MHV-68	pAB rabbit anti-MHV-68 (Hughes et al, 2010)	CB pH 6.0, 98 °C, 20 min	1:2,000, ON, 4 °C EnVision+ (HRP, Rb)[i]
Iba1	pAB rabbit anti-human Iba1[c]	CB pH 6.0, 98 °C, 20 min	1:750, 60 min, RT HRP EnVision+ (HRP, Rb)[i]
CD3	mAB rabbit anti-mouse CD3 (SP7)[d]	TE pH 9.0, 98 °C, 20 min	1:900, 60 min, 37 °C, OmniMap anti-Rb HRP[k]
CD45R	mAB rat anti-mouse CD45R (B220/RA3-6B2)[e]	CB pH 6.0, 98 °C, 20 min	1:800, 60 min, RT EnVision+ (HRP, Rat)[i]
CD31 (PECAM-1)	pAB rabbit anti-mouse PECAM-1 (M-20)[f]	TE pH 9.0, 98 °C, 20 min	1:1,000, 60 min, RT HRP EnVision+ (HRP, Rb)[i]
Laminin	pAB rabbit anti-mouse laminin[g]	Fast Enzyme[h], RT, 5 min	1:100, 60 min, RT EnVision+ (HRP, Rb)[i]
CD54 (ICAM-1)	mAB rat anti-mouse ICAM-1 (YN1/1.7.4)[g]	TE pH 9.0, 98 °C, 20 min	1:500, ON, 4 °C Rb-a-Rt IgG[m], EnVision+ (HRP, Rb)[i]
CD106 (VCAM-1)	pAB rabbit anti-mouse VCAM-1[l]	CB pH 6.0, 98 °C, 20 min	1:1,000, ON, 4 °C EnVision+ (HRP, Rb)[i]

Legend: IAV – Influenza A virus, mAB – monoclonal antibody; pAB – polyclonal antibody; CB – citrate buffer; MHV-68 – murine gammaherpesvirus-68; ON – overnight; Rb – rabbit; RT – room temperature; SARS-CoV NP – severe acute respiratory syndrome coronavirus nucleocapsid protein; TE – Tris-EDTA buffer;

Commercial providers:
[a]Rockland Immunochemicals Inc., Limerick, USA
[b]Meridian Life Sciences Inc., Memphis, USA
[c]WAKO, Osaka, Japan
[d]Spring Bioscience Corp., Ventana Medical Systems, Tucson, USA
[e]BD Pharmingen, Franklin Lakes, USA
[f]Santa Cruz Biotechnology Inc., Dallas, Text, USA
[g]Abcam, Cambridge, UK
[h]Zytomed Systems GmbH, Bargteheide, Germany
[i]Agilent Dako, Glastrup, Denmark
[k]Ventana Medical Systems Inc., Tucson, USA
[l]Invitrogen, Waltham, USA

[m]Vector Laboratories, Newark, USA

Supplemental Tables S2. Detailed results of the statistical analysis following the morphometric analysis of VCAM-1 expression in the lungs.

A) Results of the descriptive statistics and the normality test for the control group and the virus-infected groups.

	VCAM-1			
**Groups**	**Mock infected**	**SARS-CoV-2**	**IAV**	**MHV-68**
**Number of animals**	5	5	5	5
**Mean (%)**	0.4673852	1.696103	2.681738	2.791244
**Median (%)**	0.4226505	1.484835	2.743979	2.948379
**Range (%)**	0.3297457<br>0.6192877	0.9600351<br>3.2483869	1.431528<br>3.831924	1.047582<br>4.066817
**Quantile (%) (0%, 25%, 50%, 75%, 100%)**	0.3297457<br>0.3939335<br>0.4226505<br>0.5713085<br>0.6192877	0.9600351<br>1.1173894<br>1.4848349<br>1.6698668<br>3.2483869	1.431528<br>2.364904<br>2.743979<br>3.036355<br>3.831924	1.047582<br>2.515119<br>2.948379<br>3.378320<br>4.066817
**Variance**	0.01505385	0.8329033	0.7789907	1.27826
**Standard deviation**	0.1226941	0.9126354	0.8826045	1.130601
**Shapiro-Wilk test**	W = 0.91909<br>p-value = 0.5241	W = 0.82141<br>p-value = 0.1197	W = 0.99124<br>p-value = 0.9838	W = 0.95959<br>p-value = 0.8051

Legend: IAV – Influenza A virus; MHV-68 – murine gammaherpesvirus-68; 95%CI – 95 percent confidence interval. The values for the Mean, the Median, the Range, and the Quantile are expressed as percentage of positive area (%).

B) Results of the testing for the equality of variances for the different comparison pairs.

Equality of variances (F-test)			
	**SARS-CoV-2**	**IAV**	**MHV-68**
**Mock infected**	F = 0.018074<br>p-value = 0.001869	F = 0.019325<br>p-value = 0.002129	F = 0.011777<br>p-value = 0.0008066
**SARS-CoV-2**	NA	F = 1.0692<br>p-value = 0.9498	NA
**MHV-68**	F = 1.5347<br>p-value = 0.6883	F = 1.6409<br>p-value = 0.6431	NA

Legend: IAV – Influenza A virus; MHV-68 – murine gammaherpesvirus-68.

C) Results of the Welch test for the control group – virus-infected groups comparison pairs.

Welch test			
	**SARS-CoV-2**	**IAV**	**MHV-68**
**Mock infected**	t = -2.9837<br>p-value = 0.03881	t = -5.5566<br>p-value = 0.004588	t = -4.5692<br>p-value = 0.00972

Legend: IAV – Influenza A virus; MHV-68 – murine gammaherpesvirus-68.

D) Results of the t- test for the virus-infected groups comparison pairs.

t-test		
	**SARS-CoV-2**	**IAV**
**MHV-68**	t = 1.6854<br>p-value = 0.1304	t = 0.17072<br>p-value = 0.8687
**SARS-CoV-2**	NA	t = -1.7359<br>p-value = 0.1208

Legend: IAV – Influenza A virus; MHV-68 – murine gammaherpesvirus-68.

E) Results of the testing for the power of the statistical tests for the different comparison pairs.

Power of the statistical tests			
	**SARS-CoV-2**	**IAV**	**MHV-68**
**Mock infected**	power = 0.4015531	power = 0.8643602	power = 0.8941414
**SARS-CoV-2**	NA	power = 0.2792052	NA
**MHV-68**	power = 0.3322376	power = 0.03544357	NA

Legend: IAV – Influenza A virus; MHV-68 – murine gammaherpesvirus-68.

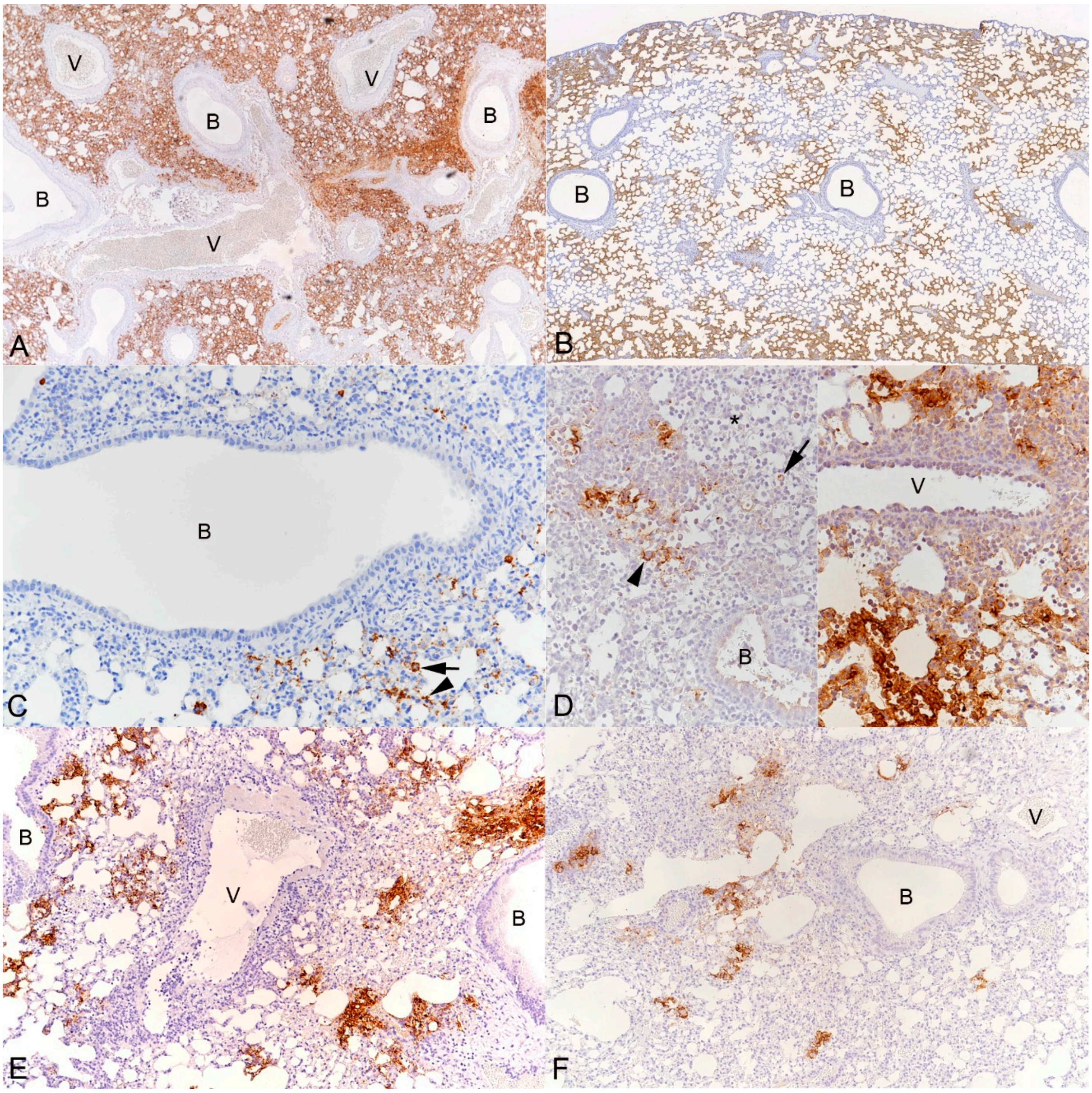

Figure S1. Viral antigen expression in the lungs of mice after SARS-CoV-2 infection. **A)** K18-hACE2 mouse at 3 days post intranasal infection with SARS-CoV-2 Pango B at 10^4 PFU. There is almost diffuse viral antigen expression in alveoli. **B)** K18-hACE2 mouse at 3 days post intranasal infection with SARS-CoV-2 Alpha at 10^3 PFU. Multifocal extensive and coalescing large areas with viral antigen expression in alveoli. **C)** BALB-C mouse at 4 days post intranasal infection with SARS-CoV-2 Beta at $2x10^5$ PFU. There are a few small patches of alveoli with positive type I (arrowhead) and II (arrow) pneumocytes. **D)** K18-hACE2 mouse at 7 days post intranasal infection with SARS-CoV-2 Pango B at 10^4 PFU. Left: Focal area with patches of alveoli with positive type I and II (arrowhead) pneumocytes and desquamed positive cells (arrow), close to an area of inflammation. Right: Large patch of strongly positive alveoli adjacent to a muscular vein with inflammatory infiltrates. **E)** K18-hACE2 mouse at 7 days post intranasal infection with SARS-CoV-2 Delta at 10^3 PFU. Patches of strongly positive alveoli adjacent to a muscular vein (V) with inflammatory changes. **F)** K18-hACE2 mouse at 7 days post intranasal infection with SARS-CoV-2 Omicron BA.1 at 10^3 PFU. Moderate number of disseminated small patches of alveoli with

positive type I and II pneumocytes. There is no evidence of viral antigen expression in respiratory epithelial cells in bronchioles in any animal (A-F). B: bronchiole; V: muscular vein. Immunohistochemistry, hematoxylin counterstain.

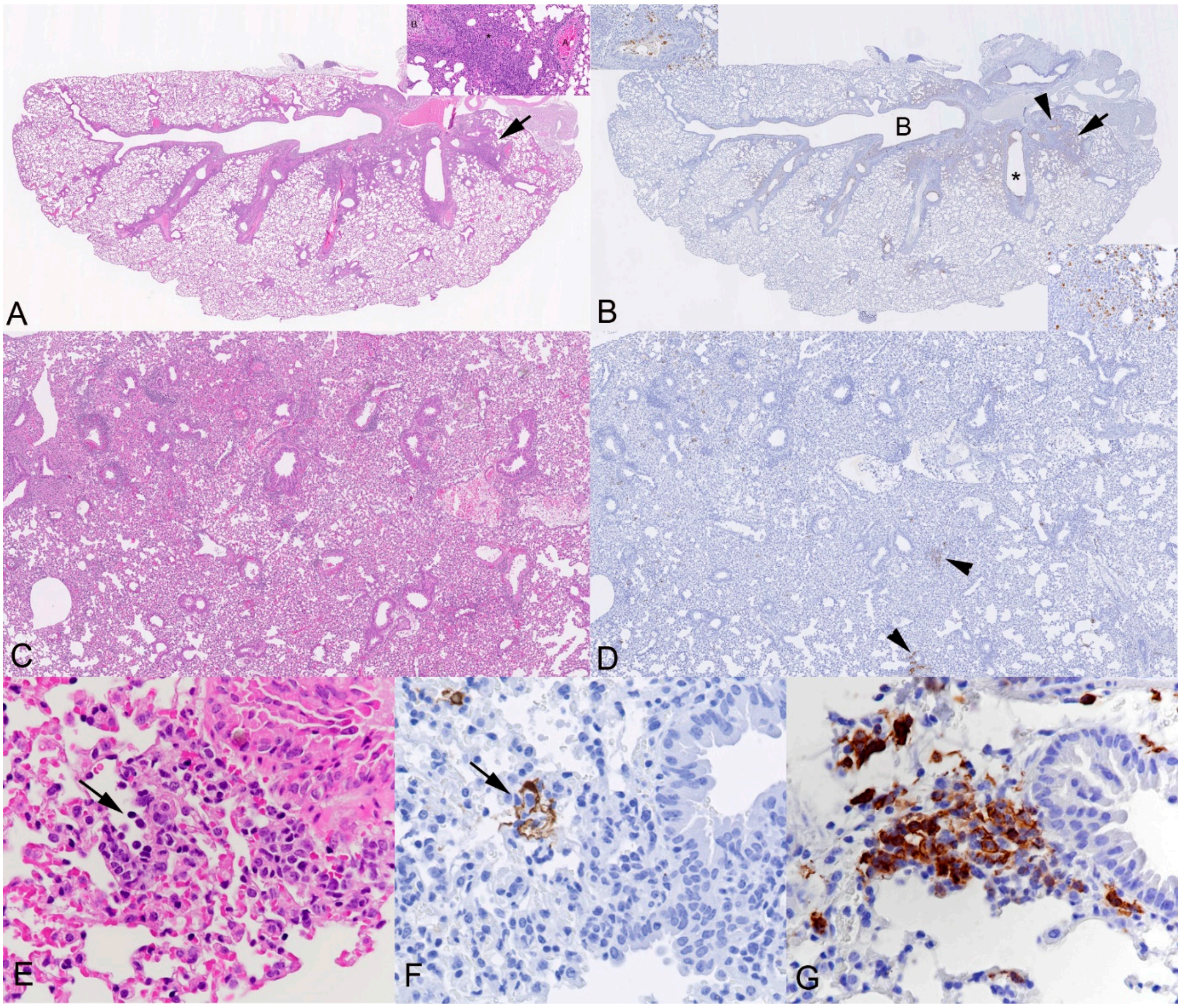

Figure S2. Histological changes and viral antigen expression in the lungs of mice infected with IAV and MHV-68. **A, B)** C57/Bl6 mouse at 5 days post intranasal infection with IAV X31 at 10^3 PFU. **A)** There is multifocal degeneration and sloughing of bronchiolar epithelial cells (arrowheads; B in inset indicates affected bronchiole). Mild multifocal increase in interstitial cellularity (arrow; * in inset) with activation of type II pneumocytes and occasional degenerate and sloughed off alveolar epithelial cells is also seen. Inset: higher magnification of area indicated by the arrow (A: artery). **B)** Viral antigen expression is seen in bronchiolar epithelial cells (* and inset top left) and within the parenchyma adjacent to infected bronchioles (arrow and inset bottom right). B: bronchiole. Inset at top left: higher magnification of bronchiole indicated by the arrowhead; inset at bottom right: higher magnification of parenchyma with infected cells, as indicated by the arrow. **C-G)** C57/Bl6 mouse at 5 days post intranasal infection with MHV-68 at 4×10^5 PFU. **C, D)** The lung exhibits moderate multifocal perivascular leukocyte infiltration (C) and small focal areas of viral antigen expression in alveolar epithelial cells (D: arrowheads). **E-G)** There are also focal parenchymal mononuclear infiltrates (E: arrow) with viral antigen expression in pneumocytes (F: arrow). Staining for the monocyte/macrophage marker Iba1 confirms that the

infiltrate is predominantly comprised of macrophages (G). HE stain (A, C, E). Immunohistochemistry, hematoxylin counterstain (B, D, F, G).

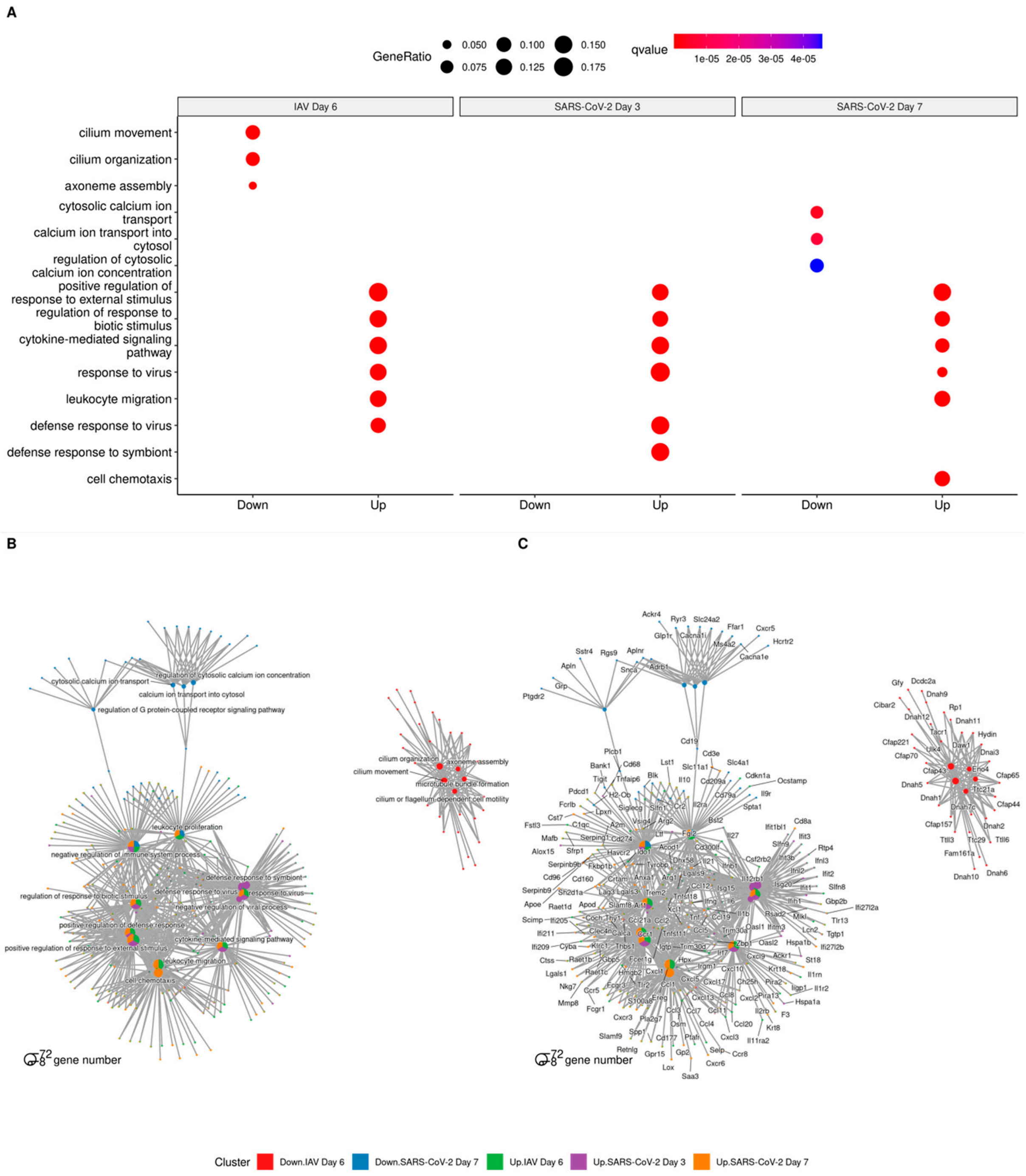

Figure S3. The top biological process terms within the dataset before extracting biological process terms associated with epithelial changes and vasculitis. **A)** The most significant biological process terms within the dataset following analysis of differentially expressed genes using clusterProfiler. Cnetplot was used to show genes associated with ontology terms highlighting biological complexity as genes belong to multiple biological process annotations. **B)** shows the category annotation, **C)** shows the gene annotation.
