# Supplementary figures and images for: The Stereotypic Response of the Pulmonary Vasculature to Respiratory Viral Infections: Findings in Mouse Models of SARS-CoV-2, Influenza A and Gammaherpesvirus Infections
Source: Viruses. 2023 Jul 27;15(8):1637. doi: 10.3390/v15081637 (PMC10458810; doi:10.3390/v15081637)

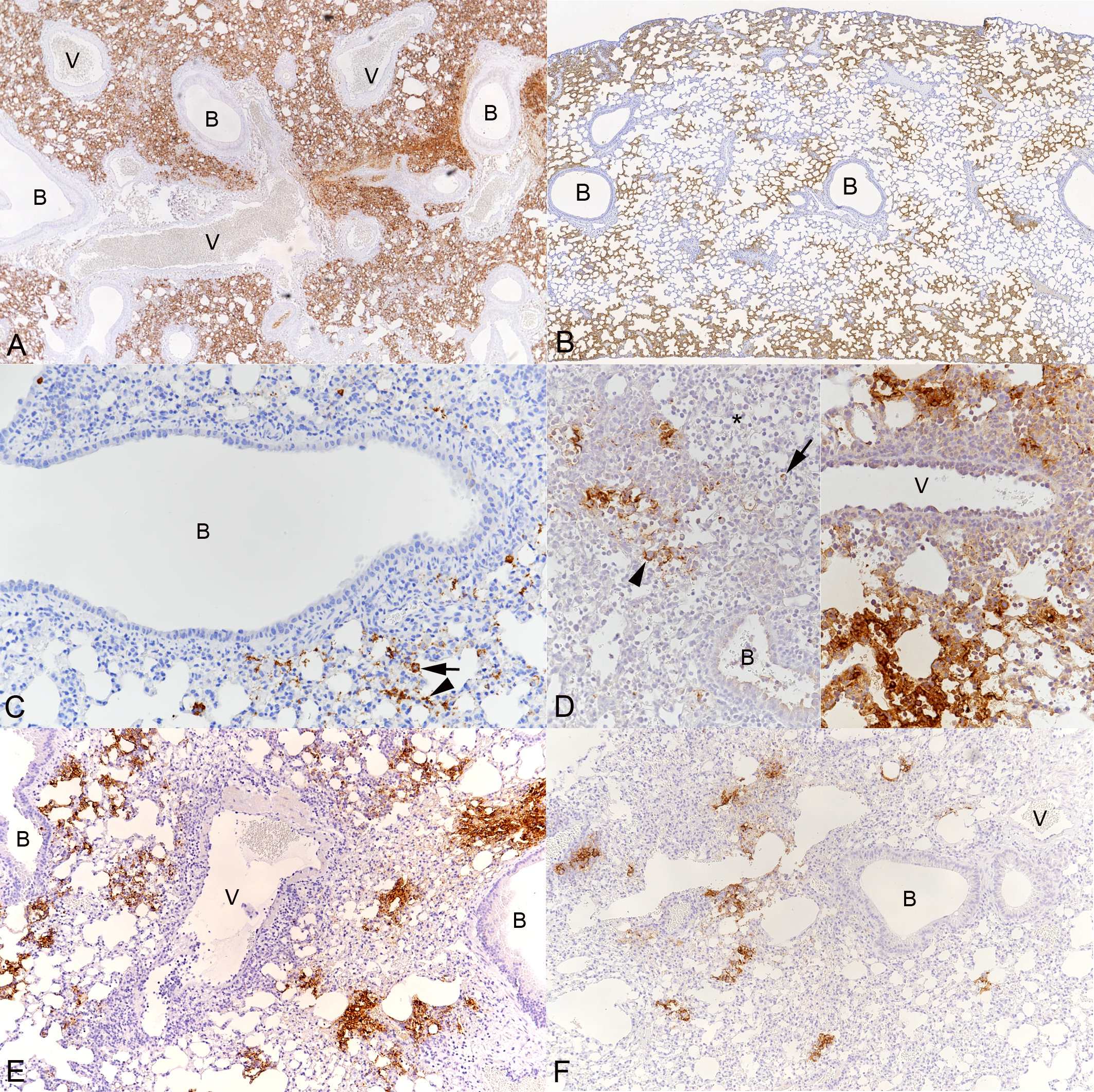

Supplement: Supplementary file 1 [file viruses-15-01637-s001.zip › Supplementary Figure S1 Assembled flat.tif]

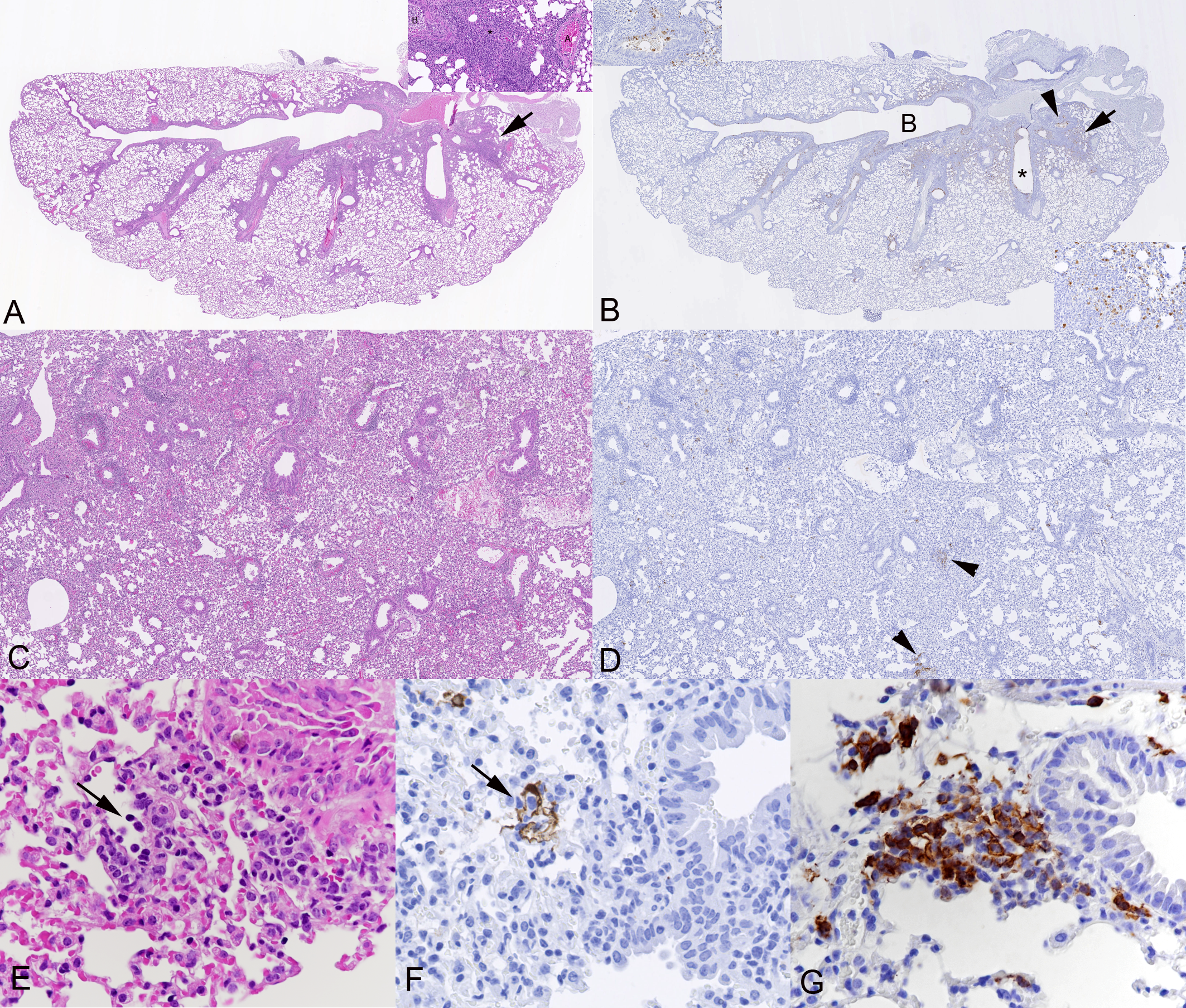

Supplement: Supplementary file 1 [file viruses-15-01637-s001.zip › Supplementary Figure S2 Assembled flat.tif]
